# The Additional Value of ^18^F-FDG PET and MRI in Patients with Glioma: A Review of the Literature from 2015 to 2020

**DOI:** 10.3390/diagnostics10060357

**Published:** 2020-05-29

**Authors:** Natale Quartuccio, Riccardo Laudicella, Antonio Vento, Salvatore Pignata, Maria Vittoria Mattoli, Rossella Filice, Alessio Danilo Comis, Annachiara Arnone, Sergio Baldari, Manlio Cabria, Angelina Cistaro

**Affiliations:** 1 Nuclear Medicine Unit, A.R.N.A.S. Ospedali Civico, Di Cristina e Benfratelli, 90127 Palermo, Italy; natale.quartuccio84@hotmail.it (N.Q.); annachiara.arnone93@gmail.com (A.A.); 2 Committee of AIMN Pediatric Study Group, 20159 Milan, Italy; 3 Nuclear Medicine Unit, Department of Biomedical and Dental Sciences and Morpho-Functional Imaging, University of Messina, 98125 Messina, Italy; riclaudi@hotmail.it (R.L.); antvento@alice.it (A.V.); pignataus@inwind.it (S.P.); filice@libero.it (R.F.); alessiodanilo.comis@gmail.com (A.D.C.); sergio.baldari@unime.it (S.B.); 4 AIMN -Italian Association of Nuclear Medicine- Young Members Working Group, 20159 Milan, Italy; 5 Department of Neurosciences, Imaging and Clinical Sciences, “G. d’Annunzio” University, 66100 Chieti, Italy; mvittoriamattoli@yahoo.it; 6 Nuclear Medicine Department, Ente Ospedaliero Ospedali Galliera, Italy, Mura delle Cappuccine, 14, 16128 Genova, Italy; manlio.cabria@galliera.it; 7 Committee of AIMN Neuroimaging Study Group, 20159 Milan, Italy; 8 Coordinator of AIMN Paediatric Study Group, 20159 Milan, Italy

**Keywords:** ^18^F-FDG, PET, MRI, glioma

## Abstract

Aim: Beyond brain computed tomography (CT) scan, Magnetic Resonance Imaging (MRI) and Positron Emission Tomography (PET) hold paramount importance in neuro-oncology. The aim of this narrative review is to discuss the literature from 2015 to 2020, showing advantages or complementary information of fluorine-18 fluorodeoxyglucose (^18^F-FDG) PET imaging to the anatomical and functional data offered by MRI in patients with glioma. Methods: A comprehensive Pubmed/MEDLINE literature search was performed to retrieve original studies, with a minimum of 10 glioma patients, published from 2015 until the end of April 2020, on the use of ^18^F-FDG PET in conjunction with MRI. Results: Twenty-two articles were selected. Combined use of the two modalities improves the accuracy in predicting prognosis, planning treatments, and evaluating recurrence. Conclusion: According to the recent literature, ^18^F-FDG PET provides different and complementary information to MRI and may enhance performance in the whole management of gliomas. Therefore, integrated PET/MRI may be particularly useful in gliomas, since it could provide accurate morphological and metabolic information in one-shoot examination and improve the diagnostic value compared to each of procedures.

## 1. Introduction

Gliomas are the most common primary intra-axial brain tumors. Gliomas originate from neuroglial cells, which forms the supportive tissue of the central nervous system (CNS). It consists of differentiated astrocytic and oligodendrocytic components. The 2016 World Health Organization (WHO) classification of CNS tumors divides gliomas in low (LGGs, grade I-II) and high-grade (HGGs, grade III-IV) levels [1,2]. Approximately, 100,000 people worldwide receive a diagnosis of glioma every year. Although comprising less than 2% of all newly diagnosed cancers, gliomas are associated with high mortality and morbidity. With a median overall survival (OS) of 14–17 months, grade IV glioma, which is formerly known as glioblastoma multiforme (GBM), is the most lethal glioma and accounts for 70–75% of all gliomas [3,4].

The main differential diagnosis of gliomas are other brain tumors, as intracranial lymphoma and metastasis, or inflammatory and infectious diseases, such as a brain abscess [5,6]. In this scenario, pre-surgical diagnostic work-up should be based on a multimodal imaging approach in order to differentiate gliomas from other brain pathologies and discriminate LGGs from HGGs [7,8]. 

Gliomas commonly recur in the next proximity of the surgical cavity [9]. Detecting recurrence in the background of the parenchymal alterations related to previous treatments, as surgery and radiotherapy (RT), is a difficult task for imaging modalities. In particular, after surgery, the common standard of care includes RT plus the alkylating agent temozolomide. Furthermore, drugs directed against the vascular endothelial growth factor (VEGF), such as Bevacizumab, have been introduced for treating recurrent GBM [10,11]. These treatments can be followed by post-treatment alterations including radiation necrosis (RN), pseudo-progression, and pseudo-response [11]. Therefore, multimodal imaging can be of paramount importance in addressing clinical management [12,13]. 

Commonly, a brain computed tomography (CT) scan is the initial imaging modality in patients with glioma, which appears as a hypodense lesion, possibly showing rim enhancement [14,15]. Despite providing important anatomical information, CT is usually followed by magnetic resonance imaging (MRI) [12], which is generally considered superior to CT in brain tumors and able to provide complementary information [16,17]. A common practice when performing an MRI is to include standard T2-weighted (T2-w), T2-fluid-attenuated inversion recovery (T2-FLAIR), T1-weighted (T1-w), T1-w contrast-enhanced (T1-CE), and diffusion weighted imaging (DWI) sequences [14,18,19]. MRI with gadolinium (Gd) contrast enhancement is considered the gold standard imaging modality for assessing brain tumors while providing information regarding location, mass effect, peritumoral edema, and contrast-enhancement. However, it cannot always distinguish gliomas from non-neoplastic lesions (e.g., brain abscesses or parasitic lesions) with a high degree of confidence or other brain tumors, such as primary central nervous system lymphoma (PCNSL) and metastases [5]. Recently, advanced “functional” MRI techniques emerged in evaluating brain tumors with the progressive introduction in the clinical setting of diffusion, perfusion-weighted, and spectroscopic sequences [12].

Positron emission tomography (PET) is a powerful molecular imaging technique, which gained increasing importance over time in neuro-oncology [20]. The main PET radiotracers available to evaluate brain tumors are carbon-11 methionine (^11^C-MET), fluorine-18 fluoroethyltyrosine (^18^F-FET), fluorine-18 fluorothymidine (^18^F-FLT), fluorine-18 fluorodihydroxyphenylalanine (^18^F-DOPA), radiolabelled choline (^11^C-choline or ^18^F-choline), and fluorine-18 fluorodeoxyglucose (^18^F-FDG). ^18^F-FDG is a glucose analog that reflects the metabolic glucose consumption of tumors, and has been one of the first PET radiotracers used in neuro-oncology [21]. Indeed, like most cancers, malignant brain tumors (and specifically HGG) demonstrate high glucose avidity. ^18^F-FDG is actively transported across the blood brain barrier (BBB) into the brain, where it is phosphorylated and trapped in tumor cells [22]. ^18^F-FDG PET imaging may play a role in the different phases of disease of glioma, even though it is currently more frequently requested to discriminate between radioncecrosis (RN) and tumor progression (TP).

Current guidelines recommend PET by using amino acids such as ^18^F-FET, ^11^C-MET, or ^18^F-DOPA for imaging in gliomas [23], which are generally recognized as superior in comparison with ^18^F-FDG in several indications for brain tumors [24]. Nevertheless, despite the limitation of a high background activity from normal brain tissue [20], ^18^F-FDG still remains the most widely available and used PET radiotracer to evaluate brain tumors and its diagnostic and prognostic value continues to be investigated. A large and consistent amount of studies have been published on the role of ^18^F-FDG in brain tumors since the 1980s [25], and the scientific ferment around it has not yet arrested in the past five years [26]. 

The aim of this narrative review is to present an update of the literature from 2015 to 2020 by discussing the scientific evidence of the utility of ^18^F-FDG- PET imaging in conjunction with MRI in patients with gliomas, and highlighting the fields in which ^18^F-FDG PET can provide advantages or complementary information to the MRI.

## 2. Methods

### 2.1. Literature Search

A comprehensive literature search of studies on the use of ^18^F-FDG PET in conjunction with MRI in patients with gliomas was performed in the PubMed/MEDLINE database. The database was interrogated from using the following search string: (“Fluorodeoxyglucose F18”[Mesh] OR “FDG”) AND (“Positron Emission Tomography Computed Tomography”[Mesh] OR “Positron-Emission Tomography”[Mesh] OR PET OR PET/MRI OR PET/MR) AND (“brain tumor” OR “brain tumour” OR “Glioma”[Mesh] OR “Optic Nerve Glioma”[Mesh] OR “Diffuse Intrinsic Pontine Glioma”[Mesh] OR “Glioma, Subependymal”[Mesh] OR “Gliosarcoma”[Mesh] OR “Astrocytoma”[Mesh] OR “Glioma of Brain, Familial” [Supplementary Concept] OR “Glioblastoma”[Mesh]) AND (“Magnetic Resonance Imaging”[Mesh] OR “Diffusion Tensor Imaging”[Mesh] OR “Diffusion Magnetic Resonance Imaging”[Mesh] OR “Multiparametric Magnetic Resonance Imaging”[Mesh] OR “Magnetic Resonance Imaging, Interventional”[Mesh] OR “Magnetic Resonance Imaging, Cine”[Mesh] OR “Fluorine-19 Magnetic Resonance Imaging”[Mesh] OR “Proton Magnetic Resonance Spectroscopy”[Mesh] OR “Magnetic Resonance Spectroscopy”[Mesh] OR “contrast-enhanced” OR “DCE” OR “Amide proton transfer”). The time interval for the literature search was from 2015 until the end of April 2020. 

### 2.2. Study Selection

Three types of studies in patients with glioma were eligible for inclusion: (1) studies using both ^18^F-FDG PET and MRI, (2) studies investigating the role of ^18^F-FDG PET/CT and MR, (3) studies employing ^18^F-FDG PET/MR. Studies omitting to report either PET or MRI findings were excluded. Only original articles with a minimum of 10 adult patients with histologically confirmed diagnosis or a radiological suspicion of glioma were selected. Only articles in English were included in the present review. Using these criteria, two researchers (N.Q. and A.V.) independently reviewed the title, the abstract, and the full text of the retrieved articles. The references of the retrieved articles were also examined to find additional relevant articles. The primary endpoints and the main findings of the articles included in this review are shown in the results section.

## 3. Results

The literature search retrieved 95 articles. Reviewing titles, abstracts, and the full text, 21 articles were selected by applying the inclusion criteria mentioned above. A further article was found screening the references [20,27,28,29,30,31,32,33,34,35,36,37,38,39,40,41,42,43,44,45,46,47]. A total of 22 studies were included in this narrative review, organized in five relevant clinical topics: (1) diagnosis and differential diagnosis, (2) grading, (3) prognosis, (4) assessment of recurrence, and (5) treatment planning and evaluation of responses to therapy. The characteristics of the studies comparing ^18^F-FDG PET imaging and MRI in patients with glioma selected in this narrative review are shown in Table 1.

### 3.1. Diagnosis and Differential Diagnosis

**Figure 1:**
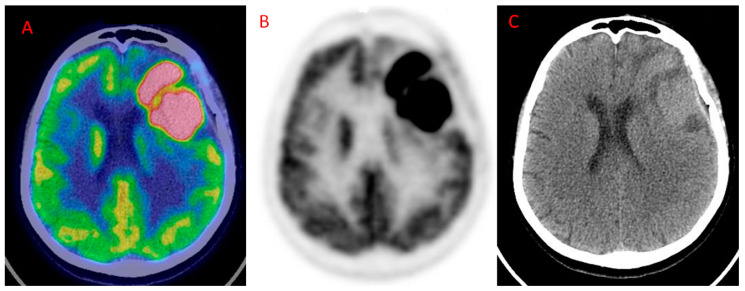
Primary Central Nervous System Lymphoma (PCNSL) in the left frontal cortex showing high ^18^F-FDG uptake. (**A**) Axial fused ^18^F-FDG PET/ CT; (**B**) ^18^F-FDG PET; (**C**): CT. (Courtesy of Dr. Cistaro).

No recent studies have investigated the role of neither ^18^F-FDG PET imaging and MRI, nor ^18^F-FDG PET/MR in patients with glioma at diagnosis. Nonetheless, Valentini and colleagues underpin the utility of combining ^18^F-FDG PET/CT and MRI for uncovering specific biological characteristics of newly diagnosed gliomas [20]. In their study, they demonstrated that the highest maximum Standardized Uptake Value (SUVmax), cerebral blood volume (CBV), Choline/Creatine (Cho/Cr), Choline/N-acetylaspartate (Cho/NAA), and Lipids/Lactate (LL) ratios were documented in the CE region. Within this region, the highest values of these parameters corresponded to the phenotype with the highest degree of malignancy and the highest molecular spectrum and stem cell potential. Conversely, the authors found a very variable range of values for apparent diffusion coefficient (ADC) and fractional anisotropy (FA) in the CE region. MRI is a very accurate diagnostic modality, especially when advanced functional techniques are added to classical morphological sequences. However, differential diagnosis between gliomas and PCNSL remains challenging. The utility of ^18^F-FDG PET imaging has, therefore, been extensively investigated in the differential diagnosis of gliomas from PCNSL (Figure 1) by means of various semiquantitative parameters [48]. Nakajima and colleagues [38], retrospectively, evaluated 23 patients with GBM and 11 patients with PCNSL, demonstrating high sensitivity (SS, 100%) and moderate specificity (SP, 73.9%) for ^18^F-FDG PET imaging using the maximum standardized uptake value [SUVmax, higher for PCNSL, optimal cutoff value = 9.35, area under the curve (AUC) = 0.925]. Furthermore, the fifth percentile value of the cumulative ADC histogram (ADC_5%_, higher for GBM) in diffusion-weighted imaging (DWI) and uncorrected CBV (higher for GBM) in dynamic susceptibility-contrast perfusion-weighted imaging (DSC-PWI) resulted in accuracy for differentiating the two malignant entities. The optimal cutoff value for ADC_5%_ was 0.68 × 10^−3^ mm^2^/s (SS = 100%, SP = 73.9%, AUC = 0.921), whereas the corresponding value for uncorrected CBV was 2.09 (SS = 90.9%, SP = 91.3%; AUC = 0.885). Another group, Yamashita and coworkers evaluated 50 patients (33 with GBM and 17 with PCNSL) who underwent ^18^F-FDG PET and MRI by assessing the diagnostic performance of ^18^F-FDG SUVmax, perfusion fraction (f), and a diffusion coefficient (D). The authors found significantly higher f_max_ (*p* < 0.001) and D_min_ (*p* < 0.0001) and significantly lower SUVmax (*p* < 0.0005) in GBM than in PCNSL. The AUC for discrimination between GBM and PCNSL were 0.756, 0.905, and 0.857 for f_max_ (optimal cut-off = 12.4%), D_min_ (optimal cut-off= 0.72 × 10^−3^ mm^2^/s), and SUVmax (optimal cut-off = 14.9), respectively [47]. The higher f_max_ and D_min_ in GBM may reflect the aberrant vasculature, whereas the high FDG uptake in PCNSL may explain the very good diagnostic performance of ^18^F-FDG PET imaging in the diagnosis of PCNSL [49].

In summary, ^18^F-FDG PET is effective for differentiating PCNSL from GBM, which suggests a potential benefit in combining PET and MRI scans.

### 3.2. Grading

^18^F-FDG uptake mirrors glucose metabolism and correlates with cell density and tumor aggressiveness [20], and may reflect better morphological features, which is better assessed by MRI, and tumor grade of gliomas. WHO grades III and IV gliomas have generally higher ^18^F-FDG uptake than LGGs, which present low glucose metabolism [23]. In this context, attention should be paid to oligodendroglioma that shows distinct pathologic and genetic features, more dense vascularization, and higher ^18^F-FDG avidity when compared to astrocytoma [50]. Therefore, the oligodendroglial component may complicate the prediction of tumor grading because higher uptake may not necessarily suggest higher proliferation [51].

A small number of studies compared ^18^F-FDG PET imaging with MRI in the assessment of grading in patients affected by gliomas, which were published in the past five years. In a recent study by Song et al., 70 patients diagnosed with primary or suspected glioma (47 cases with post-operative histological outcome), were divided into two groups. The first patient sample was examined by PET/CT and the second group was examined by MRI. SS, SP, and AC of PET/CT for grading gliomas resulted in a superior outcome to those of MRI (*p* < 0.05) [45]. However, a combination of ^18^F-FDG PET and MRI may enhance AC in the diagnosis of high-grade regions of glioma. In this regard, Shaw et al. carried out a study in 33 patients undergoing ^18^F-FDG PET/CT and Gd MRI scan. The authors documented for PET/CT an SS of 59%, an SP of 79%, a positive predictive value (PPV) of 89%, and a negative predictive value (NPV) of 55%, whereas corresponding values for MRI were SS = 77%, SP = 86%, PPV = 89%, and NPV = 71%. When ^18^F-FDG PET/CT and MRI were concordant (64% of the exams), the authors documented a combined SS of 79%, an SP of 100%, a PPV of 100%, and an NPV of 75%. The low NPV, however, should not suggest to avoid surgery in case of a negative ^18^F-FDGPET/CT alone [44]. In contrast to the commonly reported good performance in discriminating HGGs from LGGs, no encouraging results have been reported for differentiating grade III from grade II glioma using ^18^F-FDG PET and MRI. In this regard, no significant differences of ^18^F-FDG uptake, average FA, and maximum FA and minimum ADC has been reported between grade II (*n* = 23) and III (*n* = 12) in a group of 35 non-enhancing gliomas, as documented by Takano et al. [46]. The authors using the best cutoff values for the maximum tumor-to-normal tissue (T/N_max_ = 1.54) average tissue/N (T/N_ave_ = 0.61) ratios reported suboptimal AUCs for discriminating between grade II and III (AUC = 0.67 for T/N_ave;_ AUC = 0.4 for T/N_max_).

There is still a lack of comparative studies between perfusion-weighted imaging (PWI) and ^18^F-FDG PET for grading glioma. PWI allows the assessment of relative CBV (rCBV), which relates to vessel density per volume of tissue and is usually increased in HGGs [52]. ^18^F-FDG uptake and rCBV may, therefore, reflect different aspects of tumor biology [53]. In the only published study in the last five years, Sacconi et al. using the optimal cut-off values of 1.74 for rCBVmean and 4.0 for SUVmean_,_ obtained an overall SS and SP of 100% and 74% for MRI and 50% and 79.5% for PET, respectively, in discriminating HGGs from LGGs. However, this study had some important limitations. It recruited a small number of patients in different phases of disease (*n* = 20: treatment naïve = 9, post-therapy = 11). Furthermore, the histopathological results were available for only seven patients, whereas clinical and imaging follow-up was used as the standard of reference for the remaining 13 patients [40].

Amide proton transfer (APT) imaging is another promising MRI technique, which may improve the differentiation between LGGs and HGGs. This novel imaging uses off-resonance saturation pulses to identify peptides and mobile proteins in tissues [54]. In the past five years, APT was only investigated in a retrospective study by Sakata et al. in 49 newly diagnosed glioma patients, who underwent ^18^F-FDG PET/CT and MRI (including DWI) [41]. The addition of the mean APT (APT_mean_) value (extracted from a ROI placed in the most representative slice of the tumor) or minimum ADC (ADC_min_) to ^18^F-FDG uptake T/N ratio, improved discrimination of HGGs (grade III-IV) from LGGs (grade II). However, the improvement was higher when selecting the combination of T/N ratio + APT_mean_ compared to the combination APTmean + ADC_min_, as demonstrated by the net reclassification index (NRI = 0.64 vs. 0.43, respectively).

Taken altogether, the evidence from the most relevant and recent literature seems to suggest that ^18^F-FDG PET, far from being a stand-alone imaging modality in the glioma workup, may have a complementary role to enhance MRI accuracy in determining glioma grading.

### 3.3. Prognosis

Higher ^18^F-FDG uptake usually shows a correlation with worse prognosis in gliomas [50]. An interesting fact highlighted by the Lundemann et al. study the capability of PET/MR in predicting recurrence. The authors recruited 16 patients with GBM, undergoing multiparametric imaging with ^18^F-FET PET/CT and ^18^F-FDG PET/MR, including DWI and dynamic contrast-enhanced (DCE)-MRI before RT. The authors calculated the median differences of imaging parameters in recurring and non-recurring voxels for contrast-enhancing lesions (CELs), non-enhancing lesions (NELs), and normal-appearing grey and white matter within the RT target. Significant median differences were found for FA, mean diffusivity, mean transit time, extra-vascular, extra-cellular blood volume, and permeability when comparing recurring and non-recurring voxels from pre-therapy and post-therapy scans. ^18^F-FDG and ^18^F-FET uptake demonstrated the highest mutual correlation in CELs and NELs, with ^18^F-FET being the most important to predict recurrence. Nevertheless, despite a general lower predictive power of diffusion-derived parameters, fractional anisotropy resulted in the second most important parameter for recurrence probability in apparently healthy tissue [37].

The prognostic value of ^18^F-FDG PET after first-time recurrence appears under-investigated. Chiang and colleagues assessed 44 patients undergoing ^18^F-FDG PET/CT and MRI. The authors found that metabolic tumor volume and tumor cross products (obtained by multiplying the longest diameter in the axial plane by its largest perpendicular diameter) on ^18^F-FDG PET and tumor cross products on MRI were significant prognostic variables. Furthermore, combining the cross products of both PET and MRI/AC in predicting poor survival increased to 74% from 58% (using MRI alone) [29]. In another study, after evaluating 56 GBM patients with suspected disease progression on MRI (after postoperative and concurrent RT and temozolomide), a high normalized metric of metabolic activity in the residual lesion (SUV_r_), calculated as the ratio between SUVmax of the suspected region and SUVmax of contralateral background (healthy appearing white matter) demonstrated a significant association with overall survival (OS, *p* = 0.006). Patients with a SUV_r_< 1.7 had a significantly longer median survival time from PET (23.1 months) compared to patients with SUV_r_ interval of 2–2.5 (10.1 months, *p* = 0.008) or SUV_r_> 2.5 (7.5 months, *p* = 0.001). Notably, SUV_r_ did not significantly vary in patients with tumors of a different size and location at baseline MRI [36].

In summary, ^18^F-FDG PET parameters, as SUVmax, may significantly co-adjuvate MRI in evaluating prognosis in patients with glioma, especially at recurrence after post-surgical adjuvant radio-chemotherapy.

### 3.4. Assessment of Recurrence

**Figure 2:**
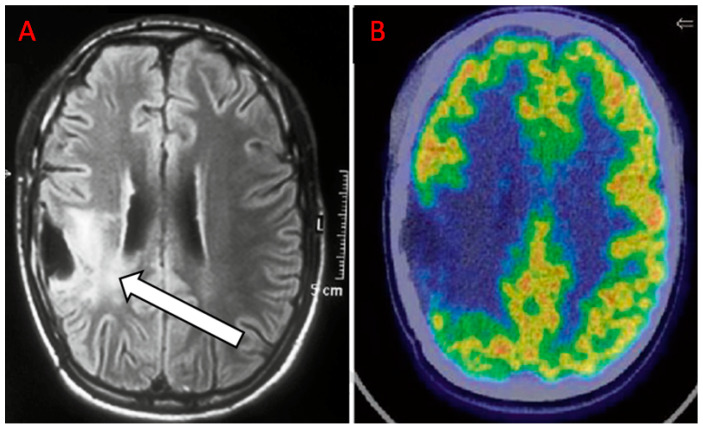
Anaplastic oligoastrocytoma treated with surgery, radiotherapy (RT), and concurrent temozolomide eight months before. (**A**) magnetic resonance imaging (MRI) shows suspicious findings for recurrence (white arrow). (**B**) the ^18^F-FDG PET/CT scan document no uptake, which is compatible with radionecrosis (RN) (Courtesy of Dr. Cistaro).

**Figure 3:**
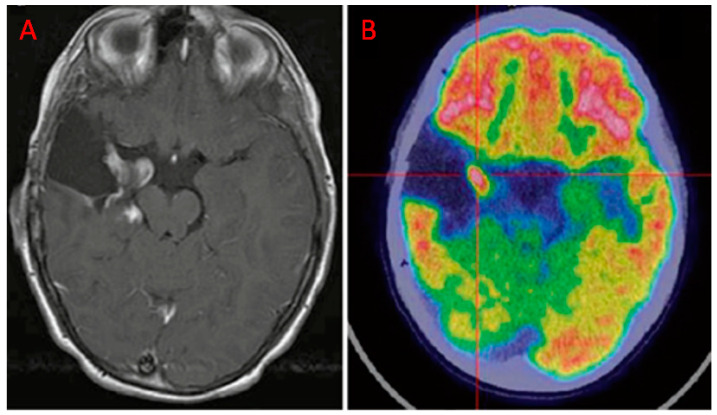
Patient with anaplastic astrocytoma, resected 2 years before, treated with temozolomide. (**A**) T1-w MRI signal alteration in the right amygdala and posterior part of the para-hippocampus. (**B**) ^18^F-FDG-avid finding, suggestive for disease recurrence (Courtesy of Dr. Cistaro).

In the follow-up of HGGs, the imaging challenge is to differentiate recurrent tumors or progressive disease from treatment-induced changes (Figure 2; Figure 3) [55].

Arora and colleagues compared ^18^F-FDG PET, Gd MRI, and found no significant difference in AC in detecting recurrence among the two modalities (82.8% and 76.6%, respectively) in 39 patients, including 29 subjects (LGGs = 15, HGGs = 14) with confirmed recurrence and 10 individuals negative for tumor recurrence, as per reference standard (clinical and/or radiological follow-up for at least six months). In a lesion-wise comparison, MRI did not detect significantly more lesions than ^18^F-FDG PET/CT (*p* = 0.14). Similarly, Iagaru et al. reported similar performance in a group of 17 patients with suspected recurrence of GBM. The patients were investigated with brain ^18^F-FDG PET/CT and Gd MRI, using a 3T MR scanner. The authors documented a comparable rate of true positive results for PET (13/15) and MR (14/15) on a per-patient analysis, as confirmed by follow-up imaging (mean 13.4 months ± 11.4) [33]. A good performance in detecting recurrence of ^18^F-FDG PET and MRI in grade I-IV oligodendroglioma (*n* = 10, 80% vs. 80%, respectively) has been reported by the study of Sharma and coworkers, who used stereotactic biopsy and clinical or imaging follow-up as standard of reference histopathology. [43]. Conversely, in low-grade astrocytomas, the authors documented a high rate of false negative results for ^18^F-FDG PET (10/22). Importantly, 5 of the 22 exams were reported as equivocal by MRI in patients, whereas ^18^F-FDG PET resulted in a true negative in four cases and a true positive (TP) in one case. ^18^F-FDG PET showed two TP results in two equivocal cases in MRI in patients with high-grade astrocytoma (*n* = 15). Few authors compared ^18^F-FDG PET imaging and DCE-MRI in detecting recurrence. A study by Hatzoglou et al., instead, included 29 gliomas (grade II = 2, grade III = 6, grade IV = 18) presenting with indeterminate enhancing brain lesions after RT [30]. The authors found that the combination of MRI and PET metrics (plasma volume ratio ≥ 2.1 and SUVratio ≥ 1.2) improved the predictive value for radiation injury compared to any individual PET and DCE-MRI metric.

MRS is another promising imaging technique, which allows the in-vivo non-invasive evaluation of the chemical composition of a scanned tissue. MRS seems as accurate as ^18^F-FDG PET in detecting glioma recurrence according to previous studies [56]. However, a direct comparison between MRS and ^18^F-FDG PET has been reported only in one study, published in 2017 by Jena and coworkers [35]. The authors aimed to discriminate glioma recurrence from treatment-induced necrosis through ^18^F-FDG PET/MR in 41 enhancing lesions (grade II = 9, III = 13, IV = 19) of 35 glioma patients comparing the diagnostic performance of rCBV, mean ADC, Cho/Cr, and maximum and mean T/N ratios by receiver operating characteristic (ROC) analysis [35]. This study confirms that PET provides complementary information to MRI since the AUC obtained combining MRI metrics and a PET parameter (mean T/N) was higher (0.935 ± 0.046) than the curve resulting only from the three MRI parameters (0.913 ± 0.053). The utility of PET/MR in differentiating tumor recurrence from RN has also been demonstrated by a study of Hojjati and colleagues, who included 24 treated GBM patients, who underwent perfusion MRI in a single examination. Specifically, the authors documented an AUC of 1.0 in a joint predictive model including r-mean ≥ 1.31 (SUVmean of the lesion / SUVmean of the contralateral background) and CBV ≥ 3.32. By contrast, a model encompassing only CBV ≥ 3.32 demonstrated a lower AUC (0.94) [32]. Seligman compared the AC of ^18^F-FDG PET and DCE-MRI in depicting glioma recurrence using a 3T PET/MRI in 41 patients with HGGs (grade III = 32, grade IV = 20) by documenting an AC of 80% and 83%, respectively, for PET and DCE-MRI. They found an association between a mutation in the receptor tyrosine kinase pathway and lower permeability (*p* = 0.038), and a trend between isocitrate dehydrogenase (IDH) mutation and lower ^18^F-FDG uptake (*p* = 0.13) [42].

Cumulatively, ^18^F-FDG PET has been shown to be helpful in differentiating glioma recurrence from RN in post-treatment MRI enhancing lesions.

### 3.5. Treatment Planning and Evaluation of Response to Therapy

PET may also help in planning surgical resection, but ^18^F-FDG uptake is influenced by cellular density and grade, as demonstrated in the study of Idegushi and coworkers. They investigated the distribution of ^18^F-FDG, ^11^C-Methionine (MET), and the hyperintense area in T2-w MR in 16 patients with glioma (grade II = 8; grade III = 8) showing absent or poor Gd enhancement. There was only partial overlap between ^18^F-FDG uptake and the contrast-enhancement area. Furthermore, the extent of delineation by ^11^C-MET PET was larger than ^18^F-FDG-PET and Gd MRI, but smaller than a T2-w abnormal signal area. In five out of six patients, tumoral tissue extracted from the ^18^F-FDG and Gd MRI positive areas presented anaplastic features, whereas tissue extracted from ^18^F-FDG and Gd MRI negative areas resulted in grade II glioma at pathological examination [34]. Based on these findings, ^11^C-MET PET appears superior to ^18^F-FDG for tumor delineation. Nevertheless, the authors suggest that ^18^F-FDG may still be useful in providing further support in the preoperative grading of gliomas, having grade II gliomas, an SUVmax, and a T/N ratio significantly lower than grade III gliomas (SUVmax 6.84 ± 1.88 grade II vs. 12.4 ± 5.28 grade III, *p* = 0.014, T/N 0.60 ± 0.15 grade II vs. 0.94 ± 0.25 grade III, *p* = 0.005).

For RT planning strategies, Hirata et al. compared the RT planning delineation based on the decoupling score (DS), derived from ^18^F-FDG and ^11^C-MET PET, with that based on T1-w and T2-w imaging in 25 patients with grade III or IV glioma [31]. DS represents the magnitude of the disrupted correlation of ^11^C-METand ^18^F-FDG, reflecting glioma cell invasion [57]. Hirata demonstrated that tumor delineation is underestimated when using T1-Gd MRI, whereas high overlap of DS and T1-Gd positively influenced survival. Furthermore, ^18^F-FDG PET guided integrated boost intensity-modulated RT (b-IMRT), which may result in a reduced dose to the normal brain when compared to standard IMRT (s-IMRT, based on T2-FLAIR MRI and ^18^F-FET PET), as demonstrated by Back and colleagues in 10 patients with anaplastic glioma carrying IDH mutation [28]. This was achieved without a significant decrease to the target volume dose despite a reduction in the prescribed dose. Few data have been published in the last five years regarding the evaluation of response to therapy by a combined ^18^F-FDG PET and MRI approach in patients with glioma. An article investigated the possibility to evaluate a response to therapy to VEGF Trap (a soluble recombinant decoy receptor inactivating extravascular and circulating VEGF) employing pre-therapy and post-therapy ^18^F-FDG PET/CT and MRI scans [39]. For monitoring the effects of this specific treatment approach, DCE-MRI appears to be the most promising imaging technique. The metrics assessed in the study were ADCmean, transfer constant (K^trans^), extravascular extracellular volume fraction (V_e_), and percent and absolute changes in SUVmax. Only the DCE-derived parameters demonstrated significant variation in a group of 12 HGG patients (median difference of K^trans^ = −41.8%, *p* < 0.02, median difference of V_e_ = −42.6%, *p* < 0.04). No significant changes were observed for SUVmax (PET/CT scan performed in 7 patients: median difference = −7.8%, *p* > 0.67), which possibly reflected the early effects of VEGF Trap on tumor vasculature without a direct impact on tumor metabolism.

Our review of the recent literature shows some evidence on the utility of ^18^F-FDG PET imaging in preoperative planning and in the evaluation of response to therapy in combination with MRI and other PET radiotracers.

## 4. Conclusions

The aim of the present review was to review the most recent studies (last five years) regarding the use of ^18^F-FDG PET in gliomas in order to clarify the advantages and disadvantages of this “elegant and evergreen” metabolic imaging in conjunction with MRI while helping the nuclear medicine community in clinical practice. The most investigated MRI techniques in the last five years in patients with glioma have been Gd MRI and DWI. Recently, although amino acid radiotracers gained a primary clinical role in PET studies of brain tumors, ^18^F-FDG still remains the most widely used and available radiotracer.

According to the recent literature, ^18^F-FDG PET provides different and complementary information to MRI and may enhance performance in differential diagnosis. In this setting, ^18^F-FDG PET appears particularly useful in the differentiation of PCNSL from GBM, potentially serving, by means of semiquantitative parameters, a confirmatory tool for MRI findings.

The combined use of ^18^F-FDG PET and MRI increase the performance in discriminating HGGs from LGGs. There are conflicting literature results to suggest the use of ^18^F-FDG PET or MRI in discriminating between grade II and III.

The combination of ^18^F-FDG PET and MRI enhance the accuracy in predicting prognosis and detecting recurrence in patients with HGG. Lastly, ^18^F-FDG PET may assist MRI in treatment planning and evaluation of response to therapy.

The development of hybrid PET/MR scanners, besides improving the diagnostic value compared to each of the individual procedures, may optimize either workflow efficiency or patient comfort. Moreover, integrated PET/MR technology will derive a combined report with features extracted from both modalities. This fact may enhance the diagnostic quality and avoid mismatched reports between radiologists and nuclear medicine physicians.

## Figures and Tables

**Table 1:** Summary of the studies included in the review.

Authors	Year	Study Design	Number of Patients	Tumor Histotype/ Glioma Grade	PET Scanner Type	MRI Technique	Main Findings
**Diagnosis and Differential Diagnosis**							
Valentini et al. [20]	2017	R	12 (48 biopsy specimens)	GBM	PET/CT	DWI, DTI, DSC-PWI, MRSI	Highest values of rCBV, Cho/Cr, Cho/NAA, proportional decrease of SUVmax with increasing distance from the CE region. At histological examination, the CE region showed maximum tumor histological malignancy and presented the maximum values of rCBV, Cho/Cr, Cho/NAA, LL and SUVmax.
Yamashita et al. [47]	2016	R	50	GBM = 33 PCNSL = 17	PET/CT	DWI, IVIM	Significantly higher fmax (*p* < 0.001) and Dmin (*p* < 0.0001) and significantly lower SUVmax (*p* < 0.0005) in GBM than in PCNSL.
Nakajima et al. [38]	2015	R	34	GBM = 23 PCNSL = 11	PET/CT	DWI, DSC-PWI	High SS (100%) and SP (73.9%) of ^18^F-FDG PET in differentiating GBM from PCNSL. Good accuracy of ADC_5%_ and uncorr
**Grading**							
Shaw et al. [44]	2019	R	33	36 histology samples: II = 11 III = 17 IV = 4 metastases = 1 benign = 3	PET/CT	Gd MRI	Combination of PET and MRI imaging enhances AC in identifying high-grade regions of glioma. PET: SS = 59%, SP = 79%, PPV = 89%, NPV = 55%. MRI: SS = 77%, SP = 86%, PPV = 89%, NPV = 71%. Combined PET and MRI: SS = 79%, SP = 100%, PPV = 100%, NPV = 75%.
Sakata et al. [41]	2018	R	49	II = 15 III-IV = 34	PET/CT	DWI, APT	Comparable AC of T/N and ADCmin and amide proton transfer in the discrimination of HGGs from LGGs. A larger increase for the diagnosis of HGGs with the combination APT + T/N compared to ADCmin + T/N.
Takano et al. [46]	2016	R	35	II = 23 III = 12	PET/CT	DTI, DWI	No satisfactory performance for average fractional anisotropy, and maximum fractional anisotropy, minimum ADC, T/Nmax and T/Nave in discriminating III from II grade.
Song et al. [45]	2016	R	70	LGG and HGG	PET/CT	Gd MRI	^18^F-FDG PET/CT performs better (in terms of SS, SP and AC) than MRI (*p* < 0.05) for identifying different grades of glioma.
Sacconi et al. [40]	2016	R	20	II = 6 III = 3 IV = 6 metastases = 2 meningioma = 2 lymphoma = 1	PET/MR	PWI	Utility of rCBVmean and SUVmean in discriminating HGGs from LGGS. rCBVmean (optimal cut-off value = 1.74): SS = 100%, SP = 74%. SUVmean_,_ (optimal cut-off value = 4.0): SS = 50%, SP = 79.5%.
**Prognosis**							
Lundemann et al. [37]	2019	P	16	GBM	PET/CT (^18^F-FET) PET/MR (^18^F-FDG)	DWI, DCE	^18^F-FDG and ^18^F-FET uptake demonstrate the highest mutual correlation in CELs and NELs, with ^18^F-FET being the most important to predict recurrence. Fractional anisotropy resulted in the second most important parameter for recurrence probability in apparently healthy tissue.
Chiang et al. [29]	2017	R	44	GBM	PET/CT	ADC	Metabolic tumor volume and tumor cross products on ^18^F-FDG PET and on MRI may serve as prognostic variables. Combining the cross products of both PET and MRI, the AC in predicting poor survival increased to 74% from 58% using MRI alone.
Leiva-Salinas et al. [36]	2017	R	56	GBM	PET/CT	Gd MRI	SUV_r_ may be a useful imaging marker to identify patients’ decreased survival after standard therapy. SUV_r_ was not influenced by tumor size and location on MRI images at diagnosis.
**Assessment of Recurrence**							
Seligman et al. [42]	2019	R	41	III = 21 IV = 20	PET/MRI	DCE	^18^F-FDG PET and DCE-MRI hold comparable AC (80% vs. 83%) in identifying tumor recurrence.
Hojjati et al. [32]	2018	R	24 (28 lesions)	GBM	PET/MRI PET/CT	DCE, DSC-PWI, DWI	The authors documented an AUC of 1.0 in a joint predictive model including r-mean ≥ 1.31 and a CBV ≥ 3.32. By contrast, a model encompassing only CBV ≥ 3.32 demonstrated a lower AUC (0.94).
Arora et al. [27]	2018	P	29	LGG = 15, HGG = 14	PET/CT	Gd MRI	On per-patient analysis, no significance difference was found between the performance of ^18^F-FDG PET/CT and MRI (AC = 82.8% vs. 76.6%) in detecting recurrence. MRI did not detect significantly more lesions than ^18^F-FDG PET/CT (*p* = 0.14).
Jena et al. [35]	2017	R	35	II = 9 III = 13 IV = 19	PET/MR	DWI, PWI, MRS	PET provides complementary information to MRI. The AUC obtained combining MRI metrics (rCBV, mean ADC, Cho/Cr) and the PET parameter (mean T/N) was higher (0.935 ± 0.046) than the curve that resulted only from the three MRI parameters (0.913 ± 0.053).
Hatzoglou et al. [30]	2016	P	29	II = 7 III = 8 IV = 18	PET/CT	DCE	The combination of a plasma volume ratio ≥ 2.1 and a SUVratio ≥ 1.2 improve the performance in distinguishing progression from radiation injury compared to individual PET and DCE metrics.
Sharma et al. [43]	2016	R	64	Low-grade astrocytoma = 22 High-grade astrocytoma = 16 Medulloblastoma = 10 Other miscellaneous brain tumors = 6	PET/CT	NR	Good performance of PET and MRI in detecting recurrence in oligodendroglioma. In low-grade astrocytomas, a high rate of false positive cases (10/22 patients) were documented for PET. Nevertheless, PET was helpful in all cases reported as equivocal (n = 5) by MRI.
Iagaru et al. [33]	2015	P	17	GBM	PET/CT	Gd MRI	Similar diagnostic performance of the two modalities for recurrent GBM (13/15 detected recurrences for PET vs. 14/15 MR).
**Treatment Planning and Evaluation of Response to Therapy**							
Idegushi et al. [34]	2018	P	16	II = 8 III = 8	PET/CT	Gd MRI, T2-w, FLAIR	^18^F-FDG PET may also help in planning surgical resection. Only partial overlap between ^18^F-FDG uptake and the contrast-enhancement area. Tissue extracted from the ^18^F-FDG and Gd MRI positive areas presented anaplastic features. Tissue extracted from ^18^F-FDG and Gd MRI negative areas resulted in grade II glioma at pathological examination.
Hirata et al. [31]	2019	P	25	III = 10 IV = 15	PET	Gd MRI, T2-w	Tumor delineation is underestimated by Gd MRI. High overlap of DS and T1-Gd positively influenced survival.
Back et al. [28]	2017	P	10	III	PET/CT	T1-w, Gd MRI, T2-w	^18^F-FDG PET guided integrated boost intensity-modulated RT (b-IMRT) that may result in a reduced dose to the normal brain when compared to standard IMRT (s-IMRT).
O’Neill et al. [39]	2016	P	12	III	PET/CT	DCE, DWI	The MRI-derived metrics (ADCmean, K^trans^, V_e)_ demonstrated significant variation in the patients (median difference of K^trans^ = −41.8%, *p* < 0.02, median difference of V_e_ = −42.6%, *p* < 0.04), possibly reflecting the early effects of VEGF trap on tumour vasculature. No systematic changes were observed for SUVmax (median difference = −7.8%, *p* > 0.67).

R: retrospective; P: prospective; N: number; GBM: glioblastoma multiforme; DWI: diffusion-weighted imaging; DSC-PWI: dynamic susceptibility-contrast perfusion-weighted imaging; MRSI: MR spectroscopic imaging; CBV: cerebral blood volume; Cho/Cr: Choline/Creatine; Cho/NAA: Choline/N-acetylaspartate; LL: Lipids/Lactate; IVIM: intravoxel incoherent motion; f: perfusion fraction; D: diffusion coefficient; SS: sensitivity; SP: specificity; AC: accuracy; ADC: apparent diffusion coefficient; Gd: gadolinium; DTI: diffusion tensor imaging; CE: contrast-enhancing; CEL: contrast-enhancing lesion; NE: non-enhancing; NEL: non-enhancing lesion; APT: amide proton transfer; T/N: tumor-to-normal tissue ratio; SUV_r_: standardized uptake value ratio (calculated as the SUVmax in the tumor relative to that in healthy white matter); AUC: area under the curve; r-mean: SUVmean of the lesion/ SUVmean of the contralateral background; FLAIR: Fluid Attenuated Inversion Recovery; T1-w: T1-weighted; T2-w: T2-weighted; DS: decoupling score (magnitude of the disrupted correlation of ^11^C-methionine and ^18^F-FDG, reflecting glioma cell invasion); K^trans^: transfer constant; V_e_: extravascular extracellular volume fraction; VEGF: vascular endothelial growth factor; VEGF Trap: a soluble recombinant decoy receptor inactivating extravascular and circulating VEGF); NR: not reported.
